# Effects of Sitting Callisthenic Balance and Resistance Exercise Programs on Cognitive Function in Older Participants

**DOI:** 10.3390/ijerph192214925

**Published:** 2022-11-13

**Authors:** Sławomir Kujawski, Agnieszka Kujawska, Mariusz Kozakiewicz, Djordje G. Jakovljevic, Błażej Stankiewicz, Julia L. Newton, Kornelia Kędziora-Kornatowska, Paweł Zalewski

**Affiliations:** 1 Department of Exercise Physiology and Functional Anatomy, Ludwik Rydygier Collegium Medicum in Bydgoszcz Nicolaus Copernicus University in Toruń, Świętojańska 20, 85-077 Bydgoszcz, Poland; 2 Department of Geriatrics, Ludwik Rydygier Collegium Medicum in Bydgoszcz Nicolaus Copernicus University in Toruń, Dębowa 3, 85-626 Bydgoszcz, Poland; 3 Translational and Clinical Research Institute, Faculty of Medical Sciences, Newcastle University, Newcastle upon Tyne NE2 4HH, UK; 4 Newcastle upon Tyne Hospitals NHS Foundation Trust, Newcastle upon Tyne NE2 4HH, UK; 5 Research Centre [CSELS], Institute for Health and Wellbeing, Faculty of Health and Life Sciences, Coventry University, and University Hospitals, Coventry CV1 5FB, UK; 6 Institute of Physical Education, Kazimierz Wielki University, Jana Karola Chodkiewicza 30, 85-064 Bydgoszcz, Poland; 7 Population Health Sciences Institute, The Medical School, Newcastle University, Framlington Place, Newcastle upon Tyne NE2 4HH, UK; 8 Laboratory of Centre for Preclinical Research, Department of Experimental and Clinical Physiology, Warsaw Medical University, 1b Banacha Street, 02-097 Warsaw, Poland

**Keywords:** physical activity, fitness, cognitive function, participation, senior, neurotrophin, aerobic capacity, mental function, hypertrophic exercise, elderly

## Abstract

Background: Exercise training programs have the potential to improve cognitive function in older subjects. However, the majority of training programs are based on aerobic modality. In the current study, the influence of 3 months programs of sitting callisthenic balance (SCB) and resistance training (RT) on cognitive functioning and the mediating role that a change in the level of neurotrophic factors and strength in older, healthy participants plays were examined. Material and methods: Global cognitive function was examined using MoCA, short-term memory using Digit Span and Delayed Matching to Sample, set shifting using Trial Making Test Part B, speed of processing simple visual stimuli using Simple Reaction Time, decision making using Choice Reaction Time, visual attention with Visual Attention Test (VAT), tests. Strength of lower and upper limbs, neurotrophin level (irisin, brain-derived neurotrophic factor (BDNF), neurotrophin 3 (NT-3), neurotrophin 4/5 (NT 4/5) were examined. Results: Improved scores in RT vs. SCB were noted in MoCA (*p* = 0.02), reaction time in SRT (*p* = 0.02), TMT B (*p* = 0.03), errors committed in CRT (*p* = 0.04) and VAT (*p* = 0.02) were observed. No significant changes in the level of neurotrophic factors were observed. Changes in upper limb strength were related to changes in the number of errors committed in the SRT (*p* = 0.03). Lower limb strength changes explained the dynamics of the number of correct answers (*p* = 0.002) and errors committed (*p* = 0.006) in VAT. Conclusions: Both SCB and RT influenced multiple cognitive domains. The RT program improved global cognitive functioning, while no improvement was noticed in the SCB group. Decision making, visual attention and global cognitive function were improved after the RT program. Set-shifting, short-term visual memory processing speed of simple visual stimuli were improved after the SCB program, while a decrease in the processing speed of simple visual stimuli was noted in the RT group. Changes in irisin were related to set-shifting and short-term memory, while in BDNF to an improvement in the processing speed of simple visual stimuli. Resistance exercise training programs could be applied to prevent age related declines of cognitive function in healthy older subjects.

## 1. Introduction

Even healthy and successful aging associates with cognitive function decline. A multicomponent physical exercise program is effective in improving cognitive function in older people [1]. However, 4-months aerobic and flexibility training with a targeted duration of 150 min/week was not found to be effective in improving the composite scores of cognitive, executive, and episodic memory function among community-dwelling older subjects [2]. The majority of training programs aimed at cognitive function improvement are based on aerobic modality. Therefore, assessment of the effect of other modalities, such as resistance exercise programs on cognitive function in older participants seems to be an important area for research.

There are few hypotheses tested on the mechanism related to the cognitive function improvement induced by the physical exercise in human-based studies. It has been shown [3] that even where studies reported an improvement in cognitive function in participants, the level of improvement did not correlate with changes in the level of functioning of other biological indicators. As Etnier et al. noted, no correlation was found between the degree of improvement in aerobic fitness and improvement in cognitive functioning [3]. In contrast, a decade later [4], other authors presented results that indicate that resistance exercise improved cognitive functioning and that this improvement was positively correlated with the increase in maximum strength in participants. It was observed that improvements in physical and cognitive function in response to physical exercise programs are positively associated, i.e., the greater increase in physical function, the better improvement in cognitive function [5]. Therefore, it is important to examine the biological correlates that could be related to this improvement in cognitive function where they have been shown to be significantly correlated with changes in the level of maximum strength. Discovering biological factors that are related to improvements in cognitive function in older people could potentially lead to the discovery of new therapeutic targets in further studies. 

To date, a range of systemic factors have been described in response to a resistance training program in older people, inter alia, overall fitness, improved blood supply to brain tissue, body composition and increased insulin sensitivity [6]. Changes specific to the brain tissue might include increased levels of brain-derived neurotrophic factor (BDNF), increased levels of neurogenesis and complexity of synaptic connections, angiogenesis, grey tissue thickness, increased levels of phosphocreatine metabolism, altered connection functionality [6]. BDNF level was positively correlated with the results of cognitive tests [7] in older participants. BDNF may also be increased as a result of undertaking resistance training programs in older subjects [8,9]. BDNF can easily penetrate the blood-brain barrier and mediate the impact of exercise on cognitive functioning [9]. Animal-based studies have shown that PGC-1α regulates the expression of *Fndc5* in neurons [10]. In turn, FNDC5 might lead to an upregulation of the expression of the BDNF [10]. As physical exercise leads to an induction of FNDC5 in the hippocampus, the PGC1a/FNDC5/BDNF pathway has been proposed as playing a role in the neuroplastic effects of physical exercise [10,11]. Animal-based studies have also shown that three months of a whole-body vibration program might lead to the modulation of FNDC5 expression and BDNF in the brain [12]. Irisin is a cleaved version of FNDC5 and has been suggested as a potential mediator of the beneficial effects of exercise on human metabolism [13]. Animal-based studies have shown that both aerobic and resistance exercise sessions might lead to an increase in the irisin level in skeletal muscles and the brain [14]. Irisin is able to cross the blood-brain barrier and eventually lead to increased BDNF expression levels in the hippocampus. It has also been shown to play a protective role in models of ischemia and neurodegenerative disorders [14]. The brain benefits induced by a physical exercise program might include the synthesis and release of neurotrophins and cytokines, neuronal autophagy as well as survival and plasticity, neurogenesis and angiogenesis and epigenetic modifications [15]. In summary, a physical exercise program might lead to the modulation of multiple potential biological factors, which presumably work in concert to lead to a delay or even reverse of the effects of aging on the brain, improve cognitive decline, and reduce the vulnerability to some neurological diseases [16]. Yau et al. [17] noted that “to be able to fully visualize changes in neurogenesis in the hippocampus by examining peripheral factors, we must examine many neurotrophins simultaneously”.

Therefore, this study will examine the impact of resistance training (RT) and sitting calisthenics balance (SCB) programs on cognitive functioning and the impact of changes in neurotrophic factors in older, healthy participants. SCB was chosen as a sham-intervention based on previous studies [18,19]. The influence of these two interventions on cognitive function tests was the primary outcome, while neurotrophic factor level changes are the secondary outcomes. Moreover, we explored the relationship between cognitive function changes with biochemical factors and physical performance and whether these might serve as correlates of cognitive function improvement. Better knowledge as to the potential correlates of cognitive function improvement in older people could lead to the discovery of new therapeutic targets in further studies.

## 2. Material and Methods

### 2.1. Setting and Enrolment

In the above lab-based 3-month, two-arm single-blind RCT, subjects over 55 years old were recruited. Participants were enrolled in studies based on advertisements in regional TV and radio, during health-promoting lectures, in Day Care Centers for the Elderly, and at various meeting groups for older people. The initial examination was conducted in the Department and Clinic of Geriatrics, Department of Hygiene, Epidemiology and Ergonomics Collegium Medicum University Hospital in Bydgoszcz, Poland. The only initial criterion for a sample was age 55+, as set in previous studies based on physical exercise in older subjects [20,21]. The study was approved by the Ethics Committee, Ludwik Rydygier Memorial Collegium Medicum in Bydgoszcz, Nicolaus Copernicus University, Torun (KB 340/2015). Written, informed consent was obtained from all participants.

The sample size calculation was conducted using the general linear mixed model power and sample size 3.0 calculator (GLIMMPSE 3.0) [22]. TMT B score was set as the primary outcome of the study, the calculation was made based on the results of previous study [23]. Results showed that to reach 0.95 power, results from 60 subjects in total had to be analyzed. Assuming a dropout rate of 14%, seventy subjects in total were assumed to initiate intervention.

Physical fitness was assessed during the physiotherapy examination. Past and current cardiac and neurological diseases, disturbances of the motor system (fractures, significant decrease of range of motion, osteoporosis) and psychiatric disorders which could significantly disturb exercises execution were excluding factors. Then, for subjects who were not rejected based on exclusion criteria, the second day of further examination was conducted.

The inclusion criteria were as follows: age 55 or older and independence of life (ability to come to the study center without additional help).

The exclusion criteria were as follows: history and current cardiologic, neurological, psychiatric, and motor disorders (including fractures, diagnosis of osteoporosis) that could significantly hinder or prevent exercise in the training program, and decreased range of motion which would prevent exercise execution.

**Figure 1:**
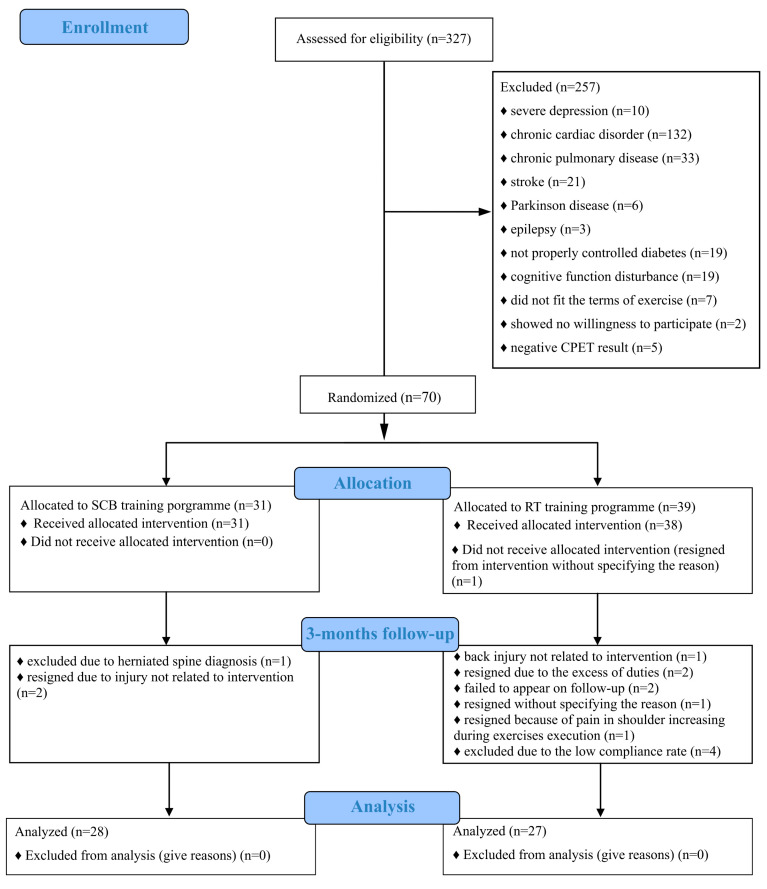
Study flow-chart CONSORT.

A total number of 327 (aged 58–91 years old) subjects were examined to select those who meet the inclusion criteria (Figure 1). Severe depression with lowered motivation was observed in 10 subjects. Chronic cardiac and chronic pulmonary disorders, which potentially would be related to need for modification of the training program occurred in 132 and 33 subjects, accordingly. A total of 6 subjects were diagnosed with Parkinson’s disease, 3 with epilepsy. There was a total of nineteen subjects with diabetes type 2 that was poorly controlled, which potentially could disturb glycemic control in response to training sessions. There were nineteen subjects with a cognitive function level that prevented fully independent functioning needed to attend organized group trainings and follow instructions (MoCA score ≤ 16 points). Seventy subjects were randomized, 28 completed the SCB, while 27 completed the RT program.

### 2.2. Depression Severity Screening and Health Self-Assessment

The 15-question version of the Geriatric Depression Scale (GDS) was used as a screening test to exclude subjects with severe depression [24]. This shorter version proved to be useful in older people with and without cognitive impairment [25]. The questions considered retrospective assessment, quality of life, current status, life activity and attitude, and other aspects. Patients who obtained a score of 12 and higher were excluded from the intervention.

### 2.3. Cognitive Assessment

To assess the global cognitive functioning of the participants, the Polish adaptation of the MoCA scale in version 7.2 was used, which is equivalent to the English version [26,27]. The MoCA was used as a screening test. The above cognitive tests were used to examine the influence of the interventions applied.

The task in Trail Making Test, Part B (TMT B), is to alternate connecting points, connecting the number with the letter, and then again with the number in ascending order (1-A-2-B, etc.) [28]. TMT B score is an indicator of executive function related to set-shifting. The score of TMT B is the time of completion of the test measured in seconds, therefore the lower the score, the better.

In the Digit Span Test (DST), the participant’s task is to repeat each sequence of numbers in the same order in which they were spoken by the examiner [29]. When the number sequence is repeated correctly, the examiner reads the next sequence. The test continues until the responder cannot repeat two strings of the same length or repeats all available strings correctly. The test result is closely related to the effectiveness of auditory attention.

In the case of the Digit Span Test Backwards (DSB), after hearing the string of numbers, the participants are asked to repeat the string in reverse order. To avoid any inaccuracies in the instructions, before proceeding with the test, each participant had to answer the question “if I say 1–2–3, what would you answer”? [29]. DSB requires storing several bits of data for a short period of time during a mental operation consisting in rotating a sequence of digits, which requires the smooth functioning of working memory [29].

To measure cognitive function the computerized battery test—Test Sprawności Operacyjnej (software version 4.6.0.44744, Speednet sp. z. o. o., more information available on: http://www.biostat.com.pl/news/nowa_aplikacja_tso_stat_-181.php, accessed on 1 April 2022) was used [30]. The following tests were included: Simple Reaction Time (SRT), Choice Reaction Time (CRT), Visual Attention Test (visual version of a match to sample—VAT) and Delayed Matching to Sample (DMS). SRT measures visual information processing speed, CRT is a decision-making test, VAT measures visual sustained attention, DMS is a test of visual form of short-term memory test. Before starting the battery test, short practice of each test was introduced for every patient. Too fast, too slow, or inadequate (wrong or double-pressed key) responses were treated as an error in this battery. A whole battery test consisted of subtests in the following order: SRT, CRT, SRT, VAT, DMS and SRT. The SRT test was repeated three times during the test period. On average, test duration was 12 min duration (i.e., between the start of whole battery test, and start of the last SRT test). Overall, there was a 2 min 20 s interval between start time of first and second SRT test. The following SRT attempts are denoted as SRT.1, SRT.2 and SRT.3.

### 2.4. Physical Fitness Assessment

The cardiopulmonary exercise test (CPET) test was performed in the presence of a physician with the Balke protocol applied (Cardiovit CS-200 Ergo-Spiro, Schiller AG, Baar, Switzerland). A short instruction of walking on a treadmill has been provided before each assessment, if needed. The test was terminated by a subject or technician at the moment of peak exertion. Otherwise, the test was ended by physician command on the basis of the American College of Sports Medicine criteria [31]. Every test was done in the same air-conditioned room with constant conditions (temperature between 20 °C and 22 °C and relative humidity of the air between 50–60%). Anaerobic threshold (AT) was determined on the basis of respiratory exchange ratio (RER) = 1 [32].

The arm curl test was performed using two types of weights: 2 kg for women and 3.5 kg for men. During the test, the subject was holding the weight in a comfortable grip, while in a sitting position on the chair with a backrest. Then, supinating during flexion was advised so that the palm of the hand faced the biceps brachii muscle at the end of the concentric phase if the initial position of the palm was directed in another way. Left- and right-hand strength were assessed separately. Mean results based on left- and right-hand scores were analyzed only. The results of this test served as upper limbs strength test.

A 30-s chair stand test was performed on the chair with a backrest. The test program includes standing from a sitting position to standing with full extension in knees and hips, without pushing off with the arms. The test score was the number of repetitions that consisted of standing and sitting phases performed in 30 s. The results of this test served as a lower limbs strength test.

### 2.5. Body Composition Analysis

To measure body composition changes, a multi-frequency bioelectrical impedance analyzer (Tanita MC-180MA Body Composition Analyzer, Tanita UK Ltd.) was applied [33]. The methodology of this examination was described previously [34]. In brief, body composition analysis was based on bioelectrical impedance signal. Body mass in kilograms was measured and the weight of Bone Mass (kg), Fat Mass (kg), and Lean Mass were predicted as well as Visceral Fat level.

### 2.6. Assessment of Biochemical Parameters

Peripheral blood was drawn from a vein in the arm 3 to 4 days before the first training unit and 3 to 4 days after the last training unit provided in the program. Biochemical analyses were performed using an enzyme-linked immunosorbent assay (ELISA). Levels of irisin, brain-derived neurotrophic factor (BDNF), neurotrophin 3 (NT-3), and neurotrophin 4/5 (NT-4/5) were assessed.

### 2.7. Randomization

Participants were randomly assigned to one of two groups using a random number generator using Excel (Microsoft Corporation, Redmond, WA, USA). One of the numbers has been assigned to each patient. Then, the corresponding numbers were sorted in ascending order. Participants with lower numbers were assigned to the RT group, while subjects with higher numbers to the SCB group.

### 2.8. Training Programs

After randomization, participants were advised not to change their current behavior in terms of diet and additional physical activity. The training protocol consisted of 3 months, twice per week (Tuesday-Friday) training sessions, which was described previously [34]. Each session was supervised by research assistants. Because of the supervision of each training session, potential adverse effects might be monitored by the staff Training sessions were provided in the facility of Institute of Physical Education, Kazimierz Wielki University in Bydgoszcz, Poland.

#### 2.8.1. Resistance Training Program

In short, each circuit in the RT was composed of six exercises: quarter squats on a smith machine (with a flat bench as support behind), lateral pull-down, tight adduction, sitting shoulder press, chest flies and plank, which was the only non-machine-based exercise. As American College of Sports Medicine guidelines suggest for resistance training in older adults, first circuit consisted of 15 repetitions, the second of 12 repetitions, and the third and the fourth of 10 repetitions [35]. As the effects of RT in older people might be dose-dependent, the need of constant progress of lifted weight was reminded periodically [36]. In addition, the constant progress of the training sessions intensity was achieved also by increasing the number of circuits in a progressive manner [34]. Subjects were asked to rest in-between the exercises as much time as needed in order to be ready to lift maximum weight in the next exercise, no shorter than 45 s. A single resistance training session lasted no more than 50 min.

#### 2.8.2. Sitting Balance Callisthenic Program

The training program applied in the comparator group consisted of sitting balance callisthenic exercises based on programs applied in previous research on older people [18,19]. Most of the exercises were conducted in the sitting position on Swiss balls to decrease heart rate during the session. No additional weights (e.g., hand weights or resistance bands) were applied to any of the exercises. Sufficient rest time between the exercises was managed by the main instructor, who demonstrated the technique for the exercise to participants during their rest. The SCB session included stretching and mobility exercises, basic core-strength, and balance exercises that included tai chi–based forms. The crane and the tree pose and single leg stance (eyes opened and closed) were incorporated. A single training session initially was composed of 15 exercises. To maintain the interest of the participants, five new exercises were introduced to the training protocol, each one after three weeks of the training protocol. A single training session lasted no more than 45 min.

### 2.9. Statistical Analysis

The Shapiro–Wilk test and visual inspection of histograms were used to test the assumption of normality. Levene’s was used to test if delta values (differences in score obtained just after training program minus score during baseline) measured in RT and SCB have equal variances. To examine between-group differences in delta values (RT vs SCB) the independent-sample t test or the Mann–Whitney U test was used depending on the assumptions met. To examine the interaction term of alignment group (RT vs. SCB) * effect of the training program (time: before (baseline) vs. after (just after a physical exercise program), a linear mixed model fit by REML with *t*-tests using Satterthwaite’s method were applied with lme4 [37] and lmerTest packages in R environment [38]. Subject factors were set as a random effect and group and time as fixed effects. Package car was used to conduct a type III Anova to test significance of group*effects of training program interaction [39]. Post-hoc tests were done regardless of the results of interaction term using lsmeans [40] and multcomp [41] packages. To check if changes in secondary outcomes explain changes in cognitive function level in response to physical training, a type III Anova to test the significance of group*effect of training program*explaining variable interaction was reported if explanation potential was higher than of group*effects of training program interaction [42].

## 3. Results

A total of 27 subjects from the RT group and 28 people from the SCB group were analyzed, of which there were 2 men in each group. The age of subjects ranged from 60 to 73 years of age in the RT group and from 60 to 79 years old in the SCB group (Table 1). In general, participants were independent, undertaking physical activities related to the performing of daily tasks. None of the participants were undertaking a regular physical exercise program at the time of participation in the study. Moreover, it was advised that patients did not change their current diet and physical activity level apart from that required as part of this the study.

### 3.1. Impact of Intervention on Cognitive Functioning

**Figure 2:**
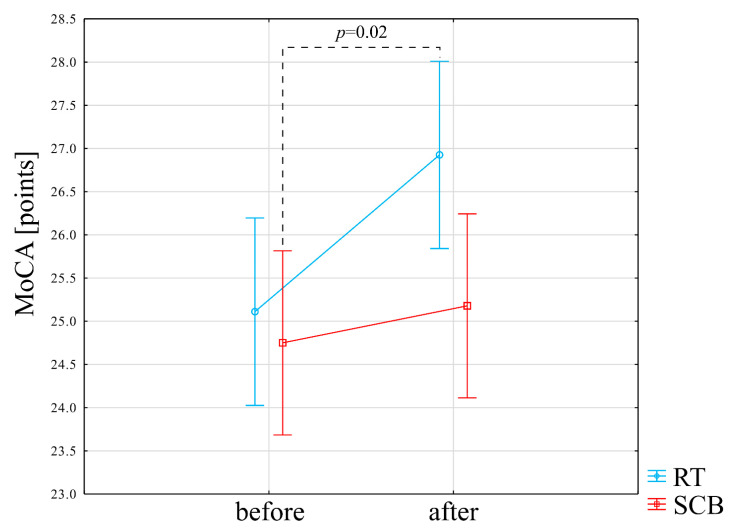
Impact of training programs on the MoCA score.

Interaction (group*time) was not significant in the case of the MoCA score (F = 1.78, *p* = 0.19). Post-hoc results indicated a significantly better result after the RT training program vs. SCB before (*p* = 0.02) (Figure 2) (Table S1).

Results from the RT group are denoted by blue lines, while SCB group results are denoted by red lines. Empty circles denote the mean value, vertical bars 95% of the Confidence Interval.

**Figure 3:**
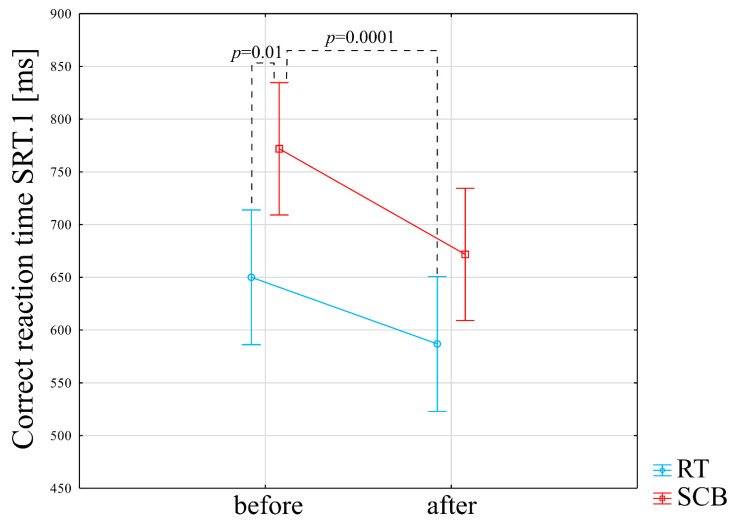
Impact of training programs on reaction time in the SRT1 score.

Interaction (group*time) was not significant in the case of reaction time in the SRT1 score (F = 0.43, *p* = 0.5). Post-hoc analysis indicated that reaction time was significantly lower (better) in the RT group both before the training program (*p* = 0.01) and in comparison, between the RT after vs. the SCB before the training program (*p* = 0.0001) (Figure 3) (Table S1).

**Figure 4:**
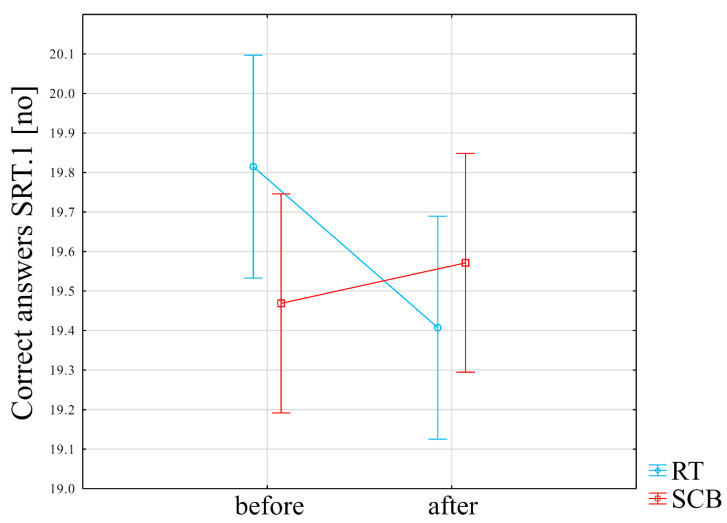
Impact of training programs on correct answers in SRT1 score.

The number of correct answers in the first attempt to SRT changed significantly in response to RT and SCB training programs (F = 4.66, *p* = 0.03). Post-hoc analysis indicated no significant differences (Figure 4) (Table S1).

Interaction (group*time) was not significant in the case of the rest of the cognitive test scores (Table S1). Post-hoc analysis indicated that the result in the RT after is significantly lower (better) than SCB before undertaking the physical exercise program in reaction time in the SRT2 (*p* = 0.02) and in the TMT B (*p* = 0.03), errors committed in the CRT (*p* = 0.04) and the VAT (*p* = 0.02).

Table S2 presents the result of the between-group comparison (SCB vs. RT) of changes in the examined scores. The number of correct answers improved in the SCB group (by a mean of 0.1) and decreased in the RT group (by mean of −0.41 after the training program), *p* = 0.02. Changes in the rest of examined cognitive function tests were not significantly different between RT vs. SCB groups (*p* > 0.05).

Table S3 presents the results of the within-group comparison (baseline vs. just after physical exercise program) in the RT group. There was a statistically significant improvement in the MoCA score (25.1 points before vs. 26.9 points after), *p* = 0.002. The reaction time in correct responses in the first attempt to the SRT was statistically significantly improved (decreases from 650 ms before to 586.81 ms after the RT program), *p* = 0.02. The number of correct answers significantly worsened in the first attempt to the SRT (19.81 correct answers before vs. 19.41 after the RT program), *p* = 0.02. Moreover, the number of errors committed in the first attempt at the SRT significantly increased (0.22 errors before vs. 0.63 after the RT program), *p* = 0.03. The reaction time in correct responses in the second attempt at the SRT was statistically significantly improved (608 ms before vs. 554.41 after), *p* = 0.02. The number of errors committed in the second attempt to the SRT was statistically significantly improved (0.52 errors before vs. 0.15 after the RT program), *p* = 0.01. The number of correct answers in the CRT significantly improved (28.81 correct answers before vs. 29.59 after the RT program), *p* = 0.04. The number of errors in the VAT decreased (11.07 errors before vs. 8.56 after the RT program), *p* = 0.04.

Table S4 presents the result of a within-group comparison (baseline vs. just after the physical exercise program) in the SCB group. There was a significant improvement (decrease in the amount of time needed for the test completion) in the result in the TMT part B (120.32 s before vs. 109.64 s after the SCB program), *p* = 0.04. In the case of the reaction time to correct responses in the first attempt to the SRT, a statistically significant improvement was observed (decrease from 779.65 ms before to 664.38 after the SCB program), *p* = 0.01. The response time to correct responses in the DMS was statistically significantly improved (decreased from 1423.20 ms before to 1266.92 after the SCB program), *p* = 0.03.

### 3.2. Impact of Intervention on Biochemical Parameters, Functional Performance and Body Composition

**Figure 5:**
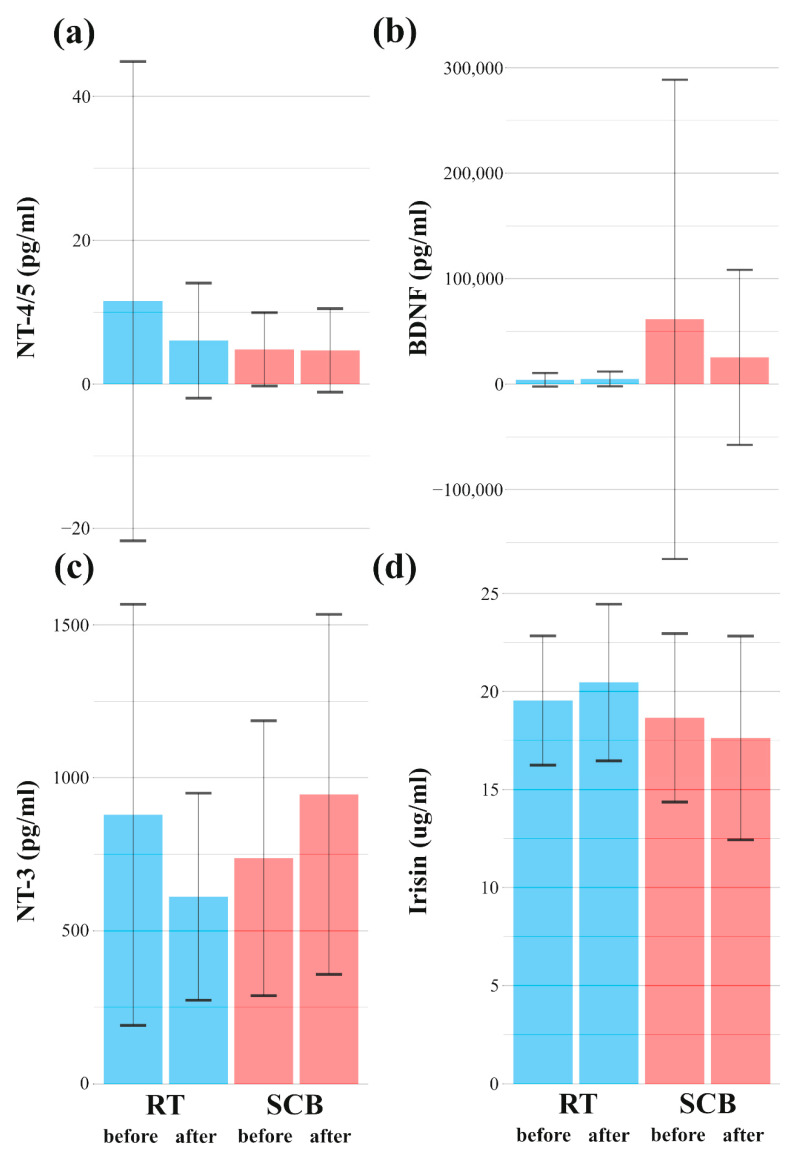
Impact of training programs on neurotrophins. Impact of training programs on: NT-4/5 (**a**), BDNF (**b**), NT-3 (**c**), Irisin (**d**).

Interaction (group*time) was not significant in the case of biochemical parameters. Moreover, post-hoc tests also revealed non-significant differences (Figure 5, Table S1).

Results from the RT group are denoted by blue bars, while the SCB group results are denoted by red bars. Whiskers denote SD.

Interaction (group*time) was not significant in the case of functional performance (Table S1). Post-hoc analysis indicated the ${\dot{V}O}_{2}$ peak was significantly higher in the RT group than in the SCB group before the training program (*p* = 0.01), higher in RT after vs. SCB before (*p* = 0.02), higher in the RT before vs. the SCB after (*p* = 0.047). In the case of post-hoc analysis of upper and lower limbs strength, a higher score was observed in the RT group after training program vs. the SCB before (*p* = 0.007 and *p* = 0.006, respectively).

Interaction (group*time) was not significant in the case of body composition. Post-hoc analysis indicated significant differences between groups in body fat mass in groups before and after the training program, RT after vs. SCB before, SCB after vs. RT before.

### 3.3. Explanation of Cognitive Function Changes

The interaction of time*group* ${\dot{V}O}_{2}$ peak was not statistically significant in explaining changes in cognitive function results. Interaction between time, group and upper limbs strength explained changes in the number of errors committed in the third attempt to the SRT (F = 4.7, *p* = 0.03). While upper limb strength improved, the number of errors committed in the third attempt at the SRT decreased in both groups (Figure S1). Interaction between time, group and lower limbs strength explained changes in the number of correct answers in the VAT (F = 10, *p* = 0.002). Lower limbs strength improvement was related to an increase in the number of correct responses in the VAT (Figure S2). Moreover, the interaction between time, group and lower limb strength explained changes in the number of errors committed in the VAT (F = 8.14, *p* = 0.006). Lower limbs strength improvement was related to a decrease in the number of errors committed in the VAT (Figure S3). Interaction between time, group and irisin explained changes in the TMT B results (F = 4.5, *p* = 0.04). Irisin changes were related to a decrease in the TMT B completion time (Figure S4). Moreover, it explained changes in errors committed in the DMS (F = 5, *p* = 0.03) (Figure S5). Interaction between time, group and the BDNF explained changes in correct reaction time in the first attempt at the SRT (F = 4.34, *p* = 0.04). BDNF changes were related to a decrease in the first attempt at the SRT reaction time (Figure S6).

## 4. Discussion

### 4.1. Impact of Resistance Training on Cognitive Function

In contrast to our results, Ansai et al. did not observe a significant improvement in cognitive functioning in participants in a 16-week resistance training program [43]. Participants were assigned to a comparator group, resistance training or multimodal training. The training program involved conducting 3 training units per week, each lasting 60 min, and exercises were performed in the range of 10–12 repetitions. In the multimodal group, a program consisting of strength, aerobic and balance exercises was conducted. Cognitive functioning was measured using the MoCA test, Clock Drawing and Verbal Fluency. No improvement in cognitive functioning was observed in any group. However, as the authors pointed out, the attrition rate in the training program was low. In the current study, in the RT group, the percentage of attendance at training units was 77%, which could probably translate into the observed improvement in the MoCA score. Furthermore, in the study of Ansai et al. [43], the mean MoCA score of subjects in the RT group was 17 and improved to 17.5 after 16 weeks of training program. In the current study, the baseline mean score in the RT group in MoCA was 25.11 and increased to 26.93 after three months of the RT program. As baseline scores of MoCA were clinically significantly higher in our sample, it might mean that participants from the current study are well characterized by better-maintained global cognitive function.

Yoon et al. [44] used an intervention based on two training groups with a training program lasting 12 weeks. The duration of a single unit was 60 min, 2 training units per week were provided. High-speed exercises were performed at highspeed using resistance straps. In the low-intensity group, exercises at a lower speed were used. Stretching was used in the control group. The duration of a single unit in the control group was 60 min and participants took part in one training unit per week. Significant improvement was observed in the MMSE and MoCA tests in both intervention groups compared to the control group. In the above study, it was also noted that there was a significant improvement in global cognitive functioning in the RT group as measured by the MoCA test. Yoon et al. [44] noted an improvement in MoCA scores from the mean of 18.29 to 24.29 points in the high-speed power training group, from 16.44 to 18.33 points in the low-speed strength training group and a decrease from 18.71 to 18.14 points in a control group consisting of balance and tone exercises. Therefore, similar to the case of comparison with the study of Ansai et al. [43], the sample examined in the study of Yoon et al. [44] was characterized by a significantly worse global cognitive function in comparison to the sample examined in the current study. It seems that a RT implemented in patients with Mild Cognitive Impairment (MCI) might lead to a decrease in atrophy of the hippocampal subfield as CA1, subiculum and dentate gyrus [45]. Therefore, further studies should examine the clinical efficacy of RT in slowing down the rate of pathological changes related to various types of dementia.

In the research by Cassilhas and others [46], two intervention groups were used, in which the length of the training program was 24 weeks, the duration of a single unit was 60 min, 3 training units per week were conducted. In one group, participants performed exercises with an intensity of 50% of repetition maximum, while in the other group, with an intensity of 80% of repetition maximum. The control group used toning training, while the duration and frequency of training units were the same as in the other groups. To assess the impact of the intervention, inter alia, Similarity Test, Digit Span Test, Corsi Pads, Toulouse-Pieron Test, Rey Figures, GDS tests were used. The maximum strength test showed no differences between the two experimental groups, but they achieved significantly better results than the control group. The high-intensity group obtained a greater increase in lean mass than the control group and achieved better results in the Digit Span Test, Corsi Pads, Similarities and Rey Figure. The lower intensity group obtained better results than the control group in the Digit Span, Corsi Blocks, Similarity, and Rey Figure. The observed improvement in the Digit Span Test is not consistent with the results of this study. In this case, for both high-intensity and moderate-intensity RT groups, Cassilhas and others [46] noted an increase in the Digit Span Forward by a mean of 0.5 points. In the current study, there was a decrease of 0.1 points in RT and a 0.25-point increase in the SCB group.

Barnes et al. used a total of four intervention groups [47]. Two groups were based on mental activity (intervention and control), two on physical activity (the training program in the intervention group was based on aerobic exercises, while the training program in the control group was based on stretching and toning exercises). No significant differences were observed between the two groups based on physical exercises in improving the functioning of verbal memory and verbal learning tests, data processing speed, visual-spatial or executive functions. Interestingly, the average standardized change due to the interventions used by the authors indicates a tendency to deteriorate results in Trail Making Test Part A and B [47]. Mean standardized changes for the TMT B were –0.018 and −0.25 for groups that included physical exercise [47]. In the current study, we have noted a decrease (i.e., improvement) in the TMT B of mean of 3.41 s in RT and by 10.68 s in SCB group. In addition, in the current study—in contrast to the Barnes et al. [47]—we have noted a significant difference in the change of processing speed of simple visual stimuli. In both the RT and SCB groups, we have noted a decrease (improvement) in reaction time on simple visual stimuli. However, we have also noted an increase (worsening) in the number of errors committed in the first attempt to SRT test in the RT group. These results could be presumably explained on the basis of a trade-off model in the case of processing speed tests; subjects have the ability to “choose” the strategy of its execution. Subjects can try to pursue faster responses, which in turn might lead to a decrease in the number of correct answers. In contrast, undertaking more effort to decrease the number of committed errors could lead to slower (i.e., longer) reaction times [48].

In the study of Fragala and others [49], the training program consisted of two training sessions per week. The intensity was measured by a scale of fatigue, so that the participants exercised at a moderate level. The duration of a single training session was not given, while the training program lasted 6 weeks. There was no intervention in the control group. Tests measuring spatial assessment and reaction time were used. Authors suggested that “clinically significant” improvement in cognitive performance was observed in both tests used. Interestingly, no changes were observed in the BDNF level. These results coincide with the observed improvement in the last two SRT approaches in the RT group in this study. However, the results from Fragala and others [49] cannot be directly compared with the current study. Fragala and others [49] used Dynavision D2 Visuomotor Training Device to measure reaction time, which engages the motor system in a different way than the computerized battery test in the current research.

Liu-Ambrose et al. [18] used two intervention groups. In both groups, the training program lasted 52 weeks and exercises were conducted in the range between 6–8 repetitions. One training group used one training unit, while the other group conducted two training units a week. The control group used balance and toning training. The TMT A, TMT B, Digit Span Test, Stroop Test and Flanker Test were used to evaluate the effects. Both training groups significantly improved their results in the Stroop test compared to the balance and toning training group. The performance of the task improved by 12.6% and 10.9%, respectively, in resistance training groups with one training unit per week and two training units per week. In addition, a statistically significant improvement in the performance of the Flanker Test was observed as a result of the intervention. This result is consistent with the observed improvement in the correctness of responses in the Choice Reaction test observed in this study, whose structure is highly similar to the Flanker Test. Direct comparison of results with the current study is rather not possible as Liu-Ambrose et al. [18] used the difference between the TMT B and A as well as in the Digit Span Test, verbal stimuli was used, compared to numbers in the current study.

Nagamatsu et al. [50] used a training program in which the training unit lasted 60 min. Training units were conducted twice a week in the range between 6–8 repetitions. The training program was 26 weeks long. The study group went through the RT program and the control group underwent a balance and toning training program whose intensity was not specified. To evaluate the effects of the training program, inter alia, the Stroop test, TMT A, and TMT B were used. Compared to the control group, the RT group significantly improved its results in the Stroop test and performing association memory tests. However, no changes in cognitive functioning were observed in the group with the training program based on balance and toning exercises. The lack of significant improvement in TMT B results is in contrast to the results observed in this study in the RT group. Data provided in the main text of Nagamatsu et al. [50] is not enough to quantitatively compare with the results obtained in the current study.

### 4.2. Relationship of Changes in Cognitive Function, Physical Performance and Neurotrophic Factors

With respect to this study, participants had the opportunity to learn a total of 26 moves during the two warm-up protocols. The warm-up protocols were identical in both groups. However, the RT group performed six exercises in the program, with all six being performed from the first training session. The SCB group learned a total of 20 exercises, while the training units in the first three weeks of the program assumed 15 exercises, and subsequent exercises were added every 3 weeks. Ultimately, SCB participants learned to do 14 exercises more than RT participants. Even a 3-month stay in an enriched environment has improved cognitive functioning in both middle-aged and older rats [51]. However, cognitive benefits in the above study seem to be related to strength gain. It can be assumed that the improvement of cognitive domains only in one of the training groups results from a biological mechanism specific for physical training with a given modality. Syed-Abdul noted a significant improvement in subjective assessment of memory and sleep as well as body recomposition (maintenance of muscle mass while reduction of fat mass) after 8 weeks of the resistance exercise program [52]. Flanagan et al. [53] observed in highly trained strength athletes changes in cortical activity measured by means of an electroencephalograph during strength exercises. This activity was higher the more intense the exercise was. Therefore, the use of neuroimaging techniques should be taken into account when designing the methodology for subsequent studies on the impact on cognitive functioning. Nevertheless, the methodology of this study fits in with the directions set by the Authors of the review, which emphasized the need for future research to determine training program variables such as intensity, duration and types of exercises that are most effective in cognitive function improvement in older subjects [54].

In line with our results, Mavros et al. concluded that the RT program resulted in improvements in cognitive function and muscle strength [4]. Changes in strength, but not in aerobic capacity were related to cognitive improvement. Other authors have noted changes in BDNF levels were mediating the effect of the walking program on task-switch performance [55]. However, in the current study, a tendency towards increased BDNF was observed after the resistance exercise program and changes in BDNF were related to the results of the simple reaction time test.

### 4.3. Limitations and Further Studies

Our study has a number of limitations. Apart from the recommendation to maintain the current diet, its characteristics were not controlled. This could be a confounding factor, at least in the case of some measured parameters such as body mass or body composition. In contrast, keeping detailed diaries on physical activity and diet could burden the participants. The reliability of subjective assessments of food consumed is low and is not free from methodological problems [56].

A further limitation of this study is the relatively small sample. However, all training sessions were led by staff, which gives an excellent opportunity to control the adherence and the proper form of exercises execution. A supervised training program is likely to bring greater health benefits than older people exercising alone at home [57]. Therefore, more research is needed into the impact of the supervised RT program on the physical functioning of older participants to translate the results into recommendations needed from a clinical perspective. Both training protocols used in the above studies included weightlifting (external or body weight) without a subjective method of assessing training intensity, which could probably interfere with the results observed. Using the Borg scale could minimize this effect. In contrast, participants in the RT group were encouraged to choose the weight to be lifted in such a way that the performance of the last repetition in each series was associated with a significant level of fatigue. Therefore, it can be estimated that despite the lack of assessment of the % of 1 repetition maximum for the exercises included in the program, participants performed exercises at the recommended intensity, adapted to the subjective possibilities. The use of more cognitive tests together with neuroimaging methods could support the process of dividing participants into subgroups due to the initial level of cognitive functioning. However, the participants were examples of successful aging, and the groups were mainly female. Further research should study the effects of regular exercise on subjects of both sexes suffering from chronic diseases, such as the respiratory or cardiovascular system.

Training combining many modalities can be seen as more attractive to participants than consisting only of resistance or aerobic exercises. The multimodal training program is recommended for older people by global organizations such as ACSM [56]. One of the insufficiently studied issues is the specifics and duration of physical exercise programs necessary to prevent functional decline or improve functionality in the spectrum of cognitive impairment and dementia.

## 5. Conclusions

Both SCB and RT influenced multiple cognitive domains. The RT program improved global cognitive functioning, while no improvement was noticed in the SCB group. Decision making, visual attention and global cognitive functioning were improved after the RT program. Set-shifting, short-term visual memory processing speed of simple visual stimuli were improved after the SCB program, while a decrease in the processing speed of simple visual stimuli was noted in the RT group.

The level of biochemical parameters did not change in response to the training program in both training groups. Improvement in peak oxygen consumption was observed in both groups, while improvement in strength was observed in the RT group only. Body fat weight decreased in both groups.

${\dot{V}O}_{2}$ peak was not related to changes in cognitive function results. The increase in upper limbs strength was related to changes in the number of errors committed in the processing of the simple visual stimuli. An increase in lower limbs strength was related to changes in the number of correct answers and errors committed in the visual attention test. Changes in irisin were related to improvement in set-shifting and errors committed in short-term memory test. BDNF change was related to improvement in the processing of simple visual stimuli.

It seems that both RT and SCB might positively influence some domains of cognitive function as well as other indicators of health and fitness in healthy, older people. However, further studies should examine the specific effects of particular modalities to describe if different physical exercise modalities (aerobic vs. resistance) might act in a complementary fashion influencing cognitive function. The role of frequency, intensity and duration of training sessions, and the intensity of the training program necessary should be assessed. Further studies should also examine potential correlates of improvement in cognitive function related to physical exercise assuming its effects to be pleiotropic.

## Figures and Tables

**Table 1:** Group’s description before physical training.

Variable (Unit)	Mean ± SD RT	Mean ± SD SCB
Age (years)	64.6 ± 4	67.7 ± 6
MoCA (points)	25.1 ± 3	24.8 ± 3
GDS (points)	2.1 ± 2	3.8 ± 3
Years of education (years)	15 ± 4	14.4 ± 4

## Data Availability

Data could be shared upon reasonable request.

## Supplementary Materials

The following supporting information can be downloaded at https://www.mdpi.com/article/10.3390/ijerph192214925/s1, Figure S1: Upper body strength and errors committed in the third attempt to SRT; Figure S2: Lower body strength and correct answers in the VAT; Figure S3: Lower body strength and errors committed in the VAT; Figure S4: Irisin and TMT B results changes; Figure S5: Irisin and errors committed in DMS changes; Figure S6: BDNF and reaction time in SRT1; Table S1: Interaction group (SCB vs. RT) * time (baseline vs. just after training program); Table S2: Between-groups (SCB vs RT) comparisons of delta values (just after training program minus baseline); Table S3: Within-group (just after training program minus baseline) comparisons in the RT group; Table S4: Within-group (just after training program minus baseline) comparisons in the SCB group.
