# Supplementary material for: Effects of Sitting Callisthenic Balance and Resistance Exercise Programs on Cognitive Function in Older Participants
Source: Int J Environ Res Public Health. 2022 Nov 13;19(22):14925. doi: 10.3390/ijerph192214925 (PMC9691233; doi:10.3390/ijerph192214925)
Supplement: Supplementary file 1 [file ijerph-19-14925-s001.zip › ijerph-1980398-supplementary.pdf]

Figure S1. Upper body strength and errors committed in the third attempt to SRT.

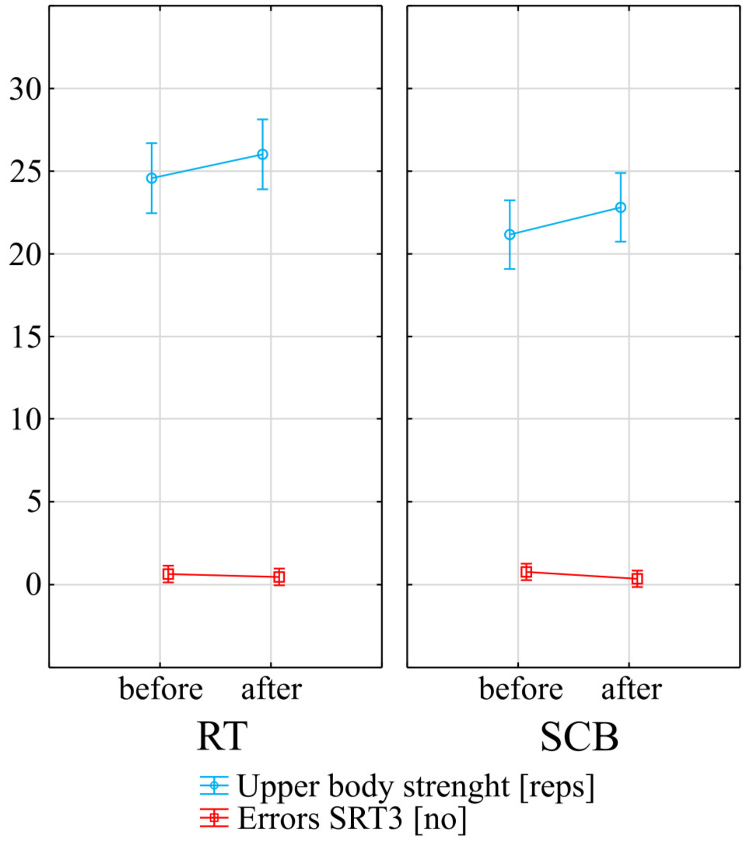

Figure S2. Lower body strength and correct answers in the VAT.

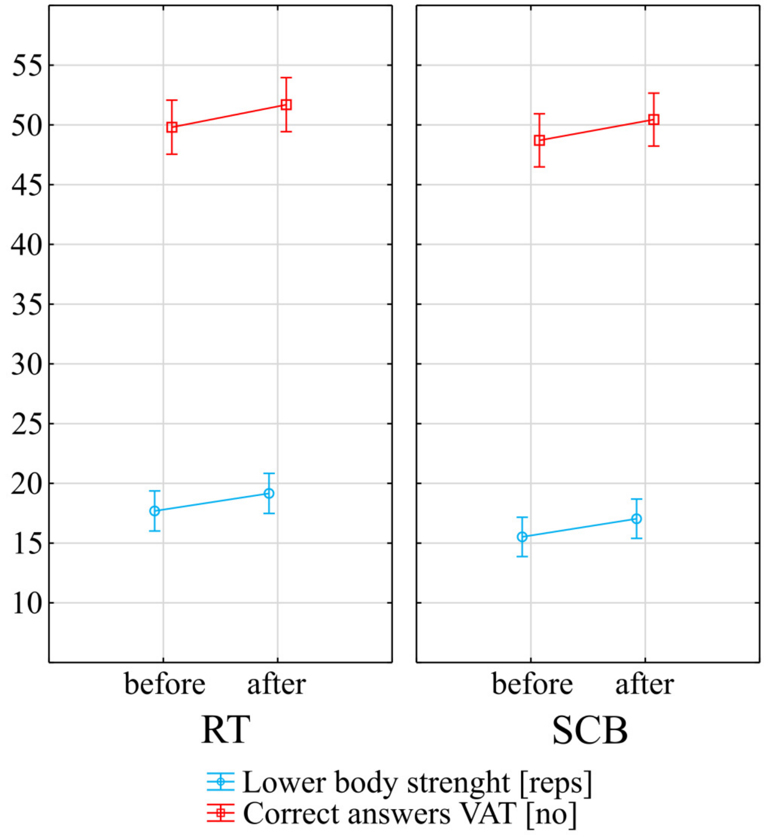

Figure S3. Lower body strength and errors committed in the VAT.

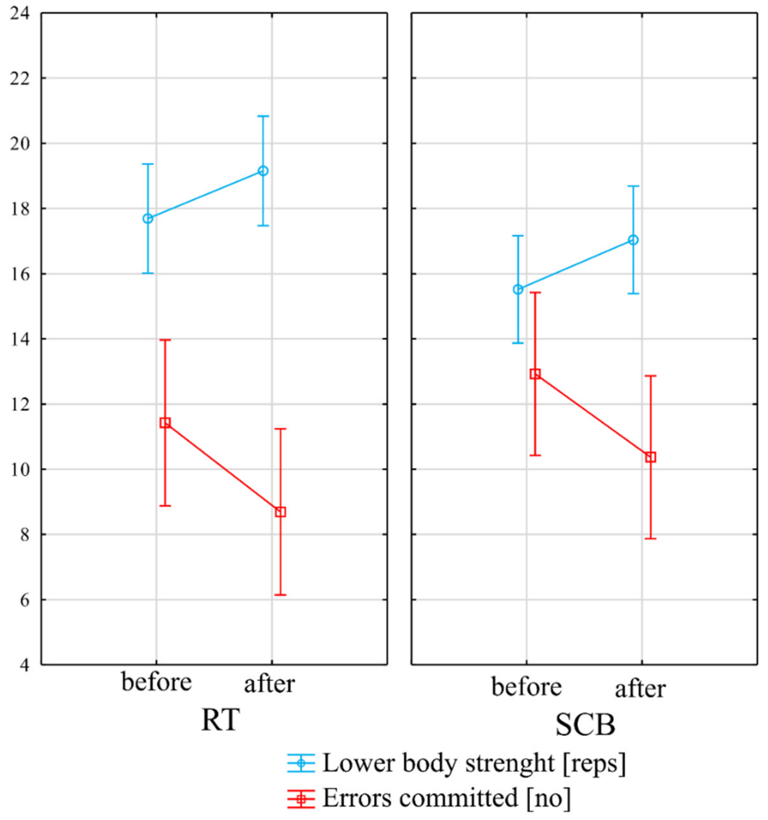

Figure S4. Irisin and TMT B results changes.

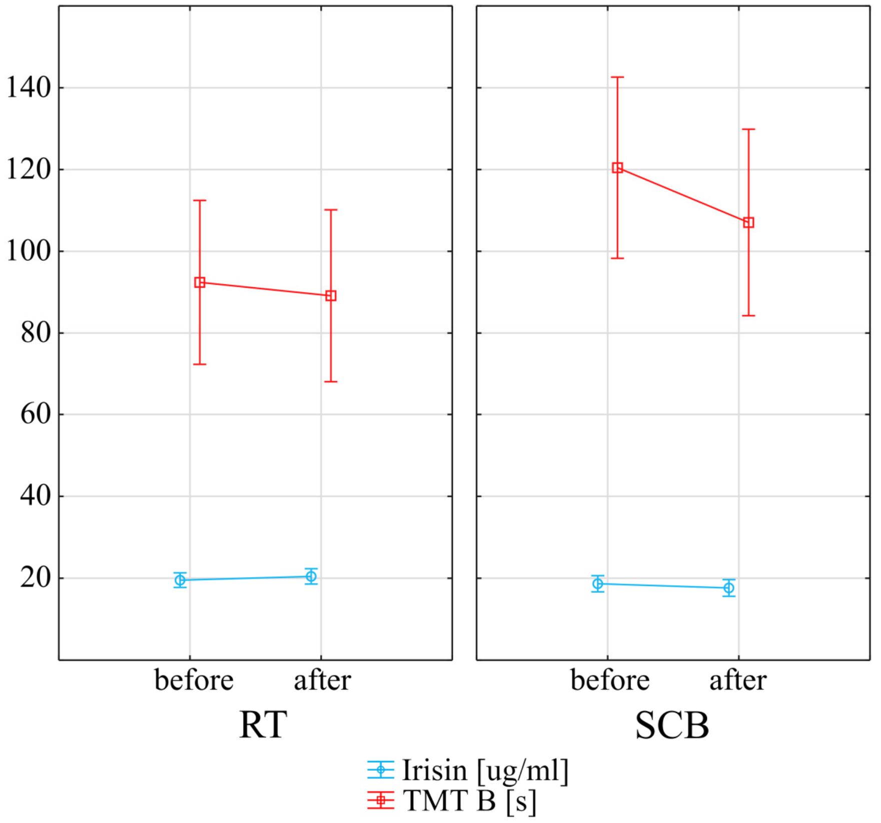

Figure S5. Irisin and errors committed in DMS changes.

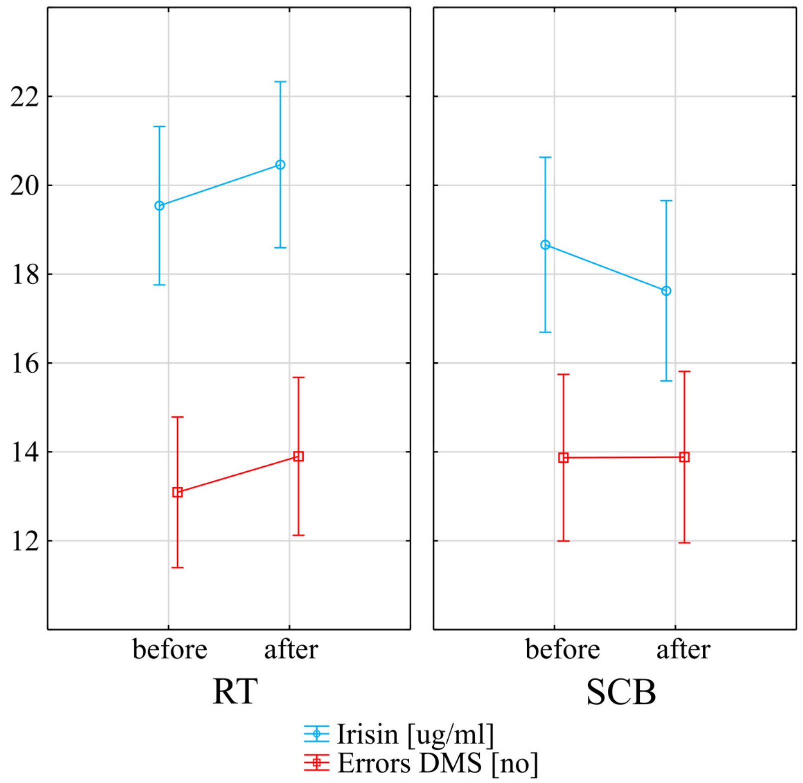

Figure S6. BDNF and reaction time in SRT1.

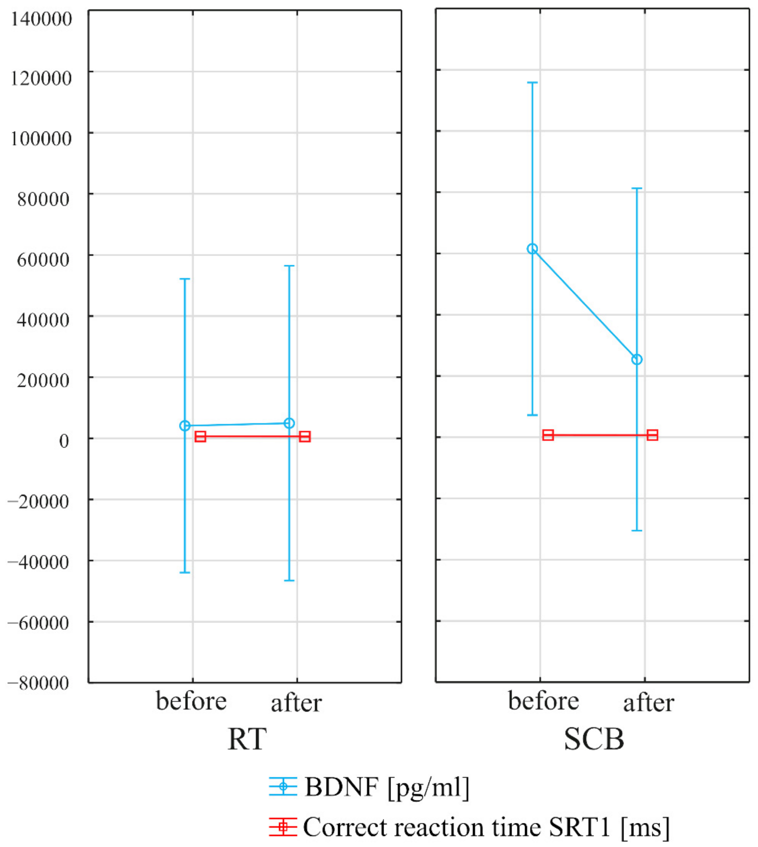

Table S1. Interaction group (SCB vs RT) * time (baseline vs just after training program).

Variable (unit)	RT before Mean±SD	RT after Mean±SD	SCB before Mean±SD	SCB after Mean±SD	group p-value	time p-value	Group*time interaction p-value
MoCA (points)	25.11±3	26.93±2.6	24.75±2.7	25.18±3	0.61	0.02	0.19
TMT B (s)	92.52±33	89.11±29	120.32±64.2	109.64±66.6	0.01	0.76	0.64
Correct reaction time SRT1 (ms)	650±124.2	586.81±107.7	779.65±253	671.75±155	0.003	0.12	0.51
Correct answers SRT1 (no)	19.81±0.4	19.41±0.8	19.46±1	19.57±0.7	0.04	0.02	0.03
Errors SRT1 (no)	0.22±0.4	0.63±0.9	1.04±2.6	0.64±1.2	0.03	0.26	0.13
Correct reaction time CRT (ms)	739.74±129.7	688.11±114.3	753.04±165.7	700.93±133.6	0.74	0.14	0.97
Correct answers CRT (no)	28.81±1.6	29.59±0.8	28.69±1.7	29.11±1.2	0.77	0.03	0.42
Errors CRT (no)	1.67±2.2	0.41±0.8	1.54±1.9	1.32±2	0.75	0.01	0.10
Correct reaction time SRT2 (ms)	608±122.0	554.41±91.4	637.69±122.5	599.96±123	0.29	0.046	0.62
Correct answers SRT2 (no)	19.56±0.6	19.85±0.5	19.81±0.5	19.75±0.7	0.07	0.03	0.06
Errors SRT2 (no)	0.52±0.8	0.15±0.5	0.38±0.9	0.39±1	0.50	0.06	0.17
Correct reaction time VAT (ms)	1699.89±225.2	1632±210.7	1764.54±220.2	1717.11±255.3	0.24	0.20	0.74
Correct answers VAT (no)	50.11±5.1	51.81±5	48.19±7.4	50±6.3	0.19	0.21	0.99
Errors VAT (no)	11.07±5.9	8.56±5.4	13.42±8.5	10.93±6.9	0.15	0.10	0.93
Correct reaction time DMS (ms)	1384.7±246.5	1352.11±266.7	1423.2±316.8	1268.18±201.7	0.63	0.62	0.21
Correct answers DMS (no)	20.78±4.6	19.81±3.1	19.6±3.5	19.61±3.5	0.19	0.27	0.45
Errors DMS (no)	12.52±4.7	13.74±3.7	14±4.0	14.36±4.6	0.14	0.22	0.55
Correct reaction time SRT3 (ms)	559.62±79.9	568.07±81.2	627.68±126.6	614.74±138.6	0.03	0.80	0.67
Correct answers SRT3 (no)	19.38±0.8	19.52±0.6	19.44±1.3	19.74±0.5	0.79	0.53	0.58
Errors SRT3 (no)	0.62±0.8	0.48±0.6	0.8±2.4	0.33±0.6	0.61	0.69	0.51
DSF (points)	6.08±2.5	6.07±1.9	5.07±1.5	5.32±1.4	0.04	0.98	0.74
DSB (points)	5.31±2.1	5.41±2.1	4.41±1.4	4.75±1	0.04	0.79	0.72
$\dot{V}O_2$ peak (l/min)	1.46±0.2	1.48±0.2	1.26±0.2	1.39±0.3	0.02	0.85	0.57
Upper body strength – mean (reps)	24.58±6.2	26.02±5	21.17±5.3	22.81±5.2	0.03	0.32	0.92
Lower body strength (reps)	17.69±4.4	19.15±4.3	15.52±4.5	17.04±4.1	0.048	0.18	0.97
Body mass (kg)	70.29±13.3	69.34±13.7	63.06±10.8	62.22±10.8	0.02	0.76	0.98
Fat Mass (kg)	25.7±10.4	23.31±8.8	16.18±5.6	14.89±5.1	0.17	0.37	0.87
Lean mass (kg)	46.33±6.9	46.66±7.2	42.58±7.8	42.9±7.9	0.04	0.86	1.00
Visceral Fat Level (units)	9.3±4.2	8.81±3.6	7.71±2.2	7.36±2.2	0.03	0.5	0.90
Bone mass (kg)	2.4±0.3	2.42±0.4	2.26±0.3	2.99±3.7	0.78	0.98	0.32
Irisin (ug/ml)	19.54±3.3	20.46±4	18.66±4.3	17.63±5.2	0.049	0.49	0.30

BDNF (pg/ml)	4128.72±6456.3	4944.91±6981.1	61520.70±227110.2	25384.29±82986	0.6	0.98	0.49
NT-3 (pg/ml)	879.08±688.3	611.36±338.3	737.15±449.3	945.95±588.4	0.07	0.11	0.06
NT-4/5 (pg/ml)	11.55±33.3	6.05±8	4.84±5.1	4.69±5.8	0.83	0.35	0.54

Table S2. Between-groups (SCB vs RT) comparisons of delta values (just after training program minus baseline).

Variable (unit)	RT<br>Mean [-95% CI; 95% CI]	SCB<br>Mean [-95% CI; 95% CI]	between group comparison<br>p-value
MoCA (points)	1.81 [0.8; 2.8]	0.43 [-0.5; 1.3]	0.05
TMT B (s)	-3.41 [-10.9; 4.1]	-10.70 [-23.7; 2.4]	0.35
Correct reaction time SRT1 (ms)	-63.20 [-115.0; -11.5]	-100.00 [-188.0; -12.0]	0.75
Correct answers SRT1 (no)	-0.41 [-0.7; -0.1]	0.10 [-0.3; 0.5]	0.02
Errors SRT1 (no)	0.41 [0.1; 0.7]	-0.37 [-1.3; 0.6]	0.15
Correct reaction time CRT (ms)	-51.60 [-105.0; 1.7]	-49.70 [-104.0; 4.7]	0.96
Correct answers CRT (no)	0.78 [0.1; 1.4]	0.39 [-0.2; 1.0]	0.43
Errors CRT (no)	-1.26 [-2.1; -0.4]	-0.20 [-1.1; 0.7]	0.2
Correct reaction time SRT2 (ms)	-53.60 [-92.5; -14.7]	-35.00 [-90.6; 20.6]	0.6
Correct answers SRT2 (no)	0.30 [0.0; 0.5]	-0.05 [-0.2; 0.1]	0.06
Errors SRT2 (no)	-0.37 [-0.6; -0.1]	0.01 [-0.3; 0.4]	0.06
Correct reaction time VAT (ms)	-67.90 [-149.0; 12.9]	-43.00 [-130.0; 44.2]	0.68
Correct answers VAT (no)	1.70 [0.0; 3.4]	1.68 [-0.5; 3.8]	0.98
Errors VAT (no)	-2.52 [-4.6; -0.4]	-2.32 [-4.9; 0.2]	0.91
Correct reaction time DMS (ms)	-32.60 [-161.0; 96.0]	-148.00 [-270.0; -25.0]	0.4
Correct answers DMS (no)	-0.96 [-2.8; 0.9]	-0.03 [-1.6; 1.6]	0.45
Errors DMS (no)	1.22 [-0.8; 3.2]	0.39 [-1.5; 2.3]	0.55
Correct reaction time SRT3 (ms)	7.25 [-30.5; 45.0]	-9.94 [-74.2; 54.4]	0.65
Correct answers SRT3 (no)	0.13 [-0.2; 0.5]	0.28 [-0.1; 0.7]	0.78
Errors SRT3 (no)	-0.13 [-0.5; 0.2]	-0.43 [-1.3; 0.4]	0.91
DSF (points)	0.01 [-0.6; 0.7]	0.23 [-0.1; 0.6]	0.57
DSB (points)	0.11 [-0.5; 0.7]	0.32 [-0.2; 0.8]	0.6
VO_2 peak (l/min)	-0.16 [-0.6; 0.2]	0.05 [-0.2; 0.3]	0.41
Upper body strength – mean (reps)	1.39 [-1.1; 3.9]	1.59 [-1.0; 4.2]	0.91
Lower body strength (reps)	1.41 [-0.3; 3.2]	1.46 [-0.1; 3.0]	0.67
Body mass (kg)	-0.95 [-1.5; -0.4]	-0.84 [-1.4; -0.2]	0.86
Fat Mass (kg)	-2.63 [-6.4; 1.2]	-1.40 [-3.1; 0.3]	0.6
Lean mass (kg)	0.33 [-0.6; 1.3]	0.33 [-0.4; 1.1]	0.88
Visceral Fat Level (units)	-0.48 [-1; 0.04]	-0.36 [-0.6; -0.09]	0.97
Bone mass (kg)	0.02 [-0.03; 0.07]	0.73 [-0.7; 2.1]	0.42

Irisin (ug/ml)	-0.76 [-2.7; 1.2]	-1.3 [-3.7; 1.1]	0.89
BDNF (pg/ml)	146 [-3367; 3658]	-24137 [-96751; 48477]	0.41
NT-3 (pg/ml)	-296 [-580; -12.3]	100 [-120; 321]	0.11
NT-4/5 (pg/ml)	-5.36 [-17.2; 6.5]	-0.27 [-2.8; 2.3]	0.86

Table S3. Within-group (just after training program minus baseline) comparisons in the RT group.

Variable (unit)	RT<br>Mean [-95% CI; 95% CI]	within group comparison<br>p-value
MoCA (points)	1.81 [0.8; 2.8]	0.002
TMT B (s)	-3.41 [-10.9; 4.1]	0.6
Correct reaction time SRT1 (ms)	-63.20 [-115.0; -11.5]	0.02
Correct answers SRT1 (no)	-0.41 [-0.7; -0.1]	0.02
Errors SRT1 (no)	0.41 [0.1; 0.7]	0.03
Correct reaction time CRT (ms)	-51.60 [-105.0; 1.7]	0.02
Correct answers CRT (no)	0.78 [0.1; 1.4]	0.05
Errors CRT (no)	-1.26 [-2.1; -0.4]	0.01
Correct reaction time SRT2 (ms)	-53.60 [-92.5; -14.7]	0.99
Correct answers SRT2 (no)	0.30 [0.0; 0.5]	0.7
Errors SRT2 (no)	-0.37 [-0.6; -0.1]	0.7
Correct reaction time VAT (ms)	-67.90 [-149.0; 12.9]	0.07
Correct answers VAT (no)	1.70 [0.0; 3.4]	0.04
Errors VAT (no)	-2.52 [-4.6; -0.4]	0.01
Correct reaction time DMS (ms)	-32.60 [-161.0; 96.0]	0.11
Correct answers DMS (no)	-0.96 [-2.8; 0.9]	0.08
Errors DMS (no)	1.22 [-0.8; 3.2]	0.04
Correct reaction time SRT3 (ms)	7.25 [-30.5; 45.0]	0.62
Correct answers SRT3 (no)	0.13 [-0.2; 0.5]	0.31
Errors SRT3 (no)	-0.13 [-0.5; 0.2]	0.24
DSF (points)	0.01 [-0.6; 0.7]	0.82
DSB (points)	0.11 [-0.5; 0.7]	0.97
VO_2 peak (l/min)	-0.16 [-0.6; 0.2]	0.85
Upper body strength – mean (reps)	1.39 [-1.1; 3.9]	0.29
Lower body strength (reps)	1.41 [-0.3; 3.2]	0.05
Body mass (kg)	-0.95 [-1.5; -0.4]	0.002
Fat Mass (kg)	-2.63 [-6.4; 1.2]	0.02
Lean mass (kg)	0.33 [-0.6; 1.3]	0.52
Visceral Fat Level (units)	-0.48 [-1; 0.04]	0.08
Bone mass (kg)	0.02 [-0.03; 0.07]	0.49
Irisin (ug/ml)	-0.76 [-2.7; 1.2]	0.62
BDNF (pg/ml)	146 [-3367; 3658]	0.75
NT-3 (pg/ml)	-296 [-580; -12.3]	0.40
NT-4/5 (pg/ml)	-5.36 [-17.2; 6.5]	0.97

Table S4. Within-group (just after training program minus baseline) comparisons in the SCB group.

Variable (unit)	SCB<br>Mean [-95% CI; 95% CI]	within group comparison<br>p-value
MoCA (points)	0.43 [-0.5; 1.3]	0.46
TMT B (s)	-10.70 [-23.7; 2.4]	0.04
Correct reaction time SRT1 (ms)	-100.00 [-188.0; -12.0]	0.01
Correct answers SRT1 (no)	0.10 [-0.3; 0.5]	0.6
Errors SRT1 (no)	-0.37 [-1.3; 0.6]	0.88
Correct reaction time CRT (ms)	-49.70 [-104.0; 4.7]	0.28
Correct answers CRT (no)	0.39 [-0.2; 1.0]	0.46
Errors CRT (no)	-0.20 [-1.1; 0.7]	0.72
Correct reaction time SRT2 (ms)	-35.00 [-90.6; 20.6]	0.78
Correct answers SRT2 (no)	-0.05 [-0.2; 0.1]	0.46
Errors SRT2 (no)	0.01 [-0.3; 0.4]	0.69
Correct reaction time VAT (ms)	-43.00 [-130.0; 44.2]	0.18
Correct answers VAT (no)	1.68 [-0.5; 3.8]	0.27
Errors VAT (no)	-2.32 [-4.9; 0.2]	0.43
Correct reaction time DMS (ms)	-148.00 [-270.0; -25.0]	0.39
Correct answers DMS (no)	-0.03 [-1.6; 1.6]	0.13
Errors DMS (no)	0.39 [-1.5; 2.3]	0.1
Correct reaction time SRT3 (ms)	-9.94 [-74.2; 54.4]	0.03
Correct answers SRT3 (no)	0.28 [-0.1; 0.7]	0.58
Errors SRT3 (no)	-0.43 [-1.3; 0.4]	0.85
DSF (points)	0.23 [-0.1; 0.6]	0.18
DSB (points)	0.32 [-0.2; 0.8]	0.24
VO_2 peak (l/min)	0.05 [-0.2; 0.3]	0.46
Upper body strength – mean (reps)	1.59 [-1.0; 4.2]	0.26
Lower body strength (reps)	1.46 [-0.1; 3.0]	0.07
Body mass (kg)	-0.84 [-1.4; -0.2]	0.01
Fat Mass (kg)	-1.40 [-3.1; 0.3]	0.1
Lean mass (kg)	0.33 [-0.4; 1.1]	0.3
Visceral Fat Level (units)	-0.36 [-0.6; -0.09]	0.02
Bone mass (kg)	0.73 [-0.7; 2.1]	0.2
Irisin (ug/ml)	-1.3 [-3.7; 1.1]	0.9
BDNF (pg/ml)	-24137 [-96751; 48477]	0.86
NT-3 (pg/ml)	100 [-120; 321]	0.42
NT-4/5 (pg/ml)	-0.27 [-2.8; 2.3]	0.81
